# Supplementary material for: New Application of the Commercially Available Dye Celestine Blue B as a Sensitive and Selective Fluorescent “Turn-On” Probe for Endogenous Detection of HOCl and Reactive Halogenated Species
Source: Antioxidants (Basel). 2022 Aug 30;11(9):1719. doi: 10.3390/antiox11091719 (PMC9495391; doi:10.3390/antiox11091719)
Supplement: Supplementary file 1 [file antioxidants-11-01719-s001.zip › antioxidants-1833452-SM-final/antioxidants-1833452-SI.pdf]

## Supplementary data

Some fluorescent HOCl probes developed since 2016 and their characteristics are presented in Table S1 (see articles [1–17]).

Table S1.Comparison of fluorescent probes for HOCl[1]

| Probe | Type of fluorescent signal | λ (nm) | LOD (nM) | Plato time (s)[2] | References |
|---|---|---|---|---|---|
| CMOS | turn-off | 405, F480↓ | 21 | 5 | [1] |
| HKOCl-3 | turn-on | 490, F527↑ | 0.33 | 60 | [2] |
| BCO | ratiometric | 372, F460↑/430 | 154 | n/a | [3] |
| BETC | turn-on | 350, F440↑ | 32 | n/a | [3] |
| FHZ | turn-on | 490, F520↑ | n/a | 60 | [4] |
| NDS | turn-on | 420, F525↑ | 105 | 16 | [5] |
| RT-1 | turn-on | 550, F587↑ | 2.18 | 180 | [6] |
| BR-O | turn-on | 610, F670↑ | 19 | 420 | [7] |
| HKOCl-4 | turn-on | 530, F577↑ | 9 | 30 | [8] |
| BRT | ratiometric | 525, F580↑/540↓ | 38 | 15 | [9] |
| BC-3 | turn-on | 620, F669↑ | 11 | 30 | [10] |
| HDI-HClO | turn-on | 440, F520↑ | 8.3 | 8 | [11] |
| HQMN | ratiometric | 370, F468↑/572↓ | 787 | 100 | [12] |
| Dcp-EPtz | turn-on | 475, F618↑ | 39 | 300 | [13] |
| RO610 | turn-on | 535, F577↑ | 29 | 30 | [14] |
| CR-Ly | ratiometric | 420, F582↑/479↓ | 12 | 50 | [15] |
| FD-301 | turn-on | 620, F686↑ | 44 | 10 | [16] |
| FN-1, FN-2 | turn-on | 490, F529↑ | 210, 230 | 300, 120 | [17] |
| CB | turn-on | 430, F590↑ | 32 | 30 | Current work |

[1]Thescopeofcomparisonincludesonlyworkspublishedbetween2016 and2021thatinvolved cell or tissue imaging of HOCl.
[2] Plateau time means the time to reach a plateau in the change in fluorescence intensity after HOCl addition.

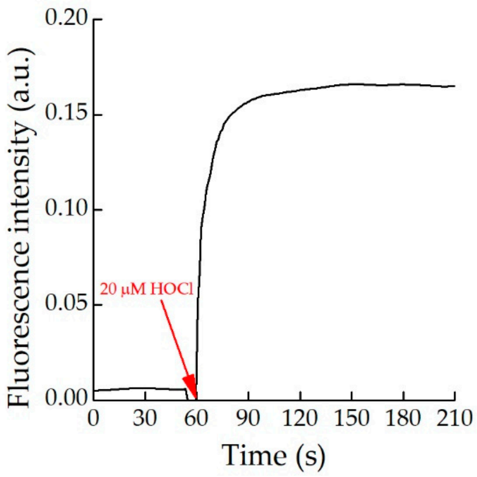


**Figure S1.** The time course of fluorescence intensity of CB (20 μM) at 590 nm ($\lambda$ex=430 nm) after treatment with 1 eqv. of HOCl (time range 0–210 s). PBS (pH7.4), T=25°C.

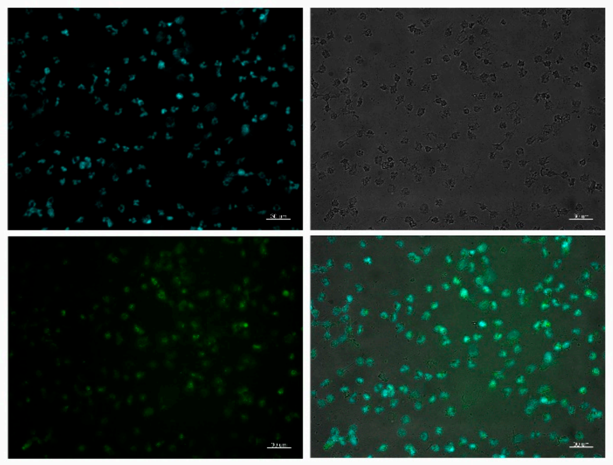

(**a**)

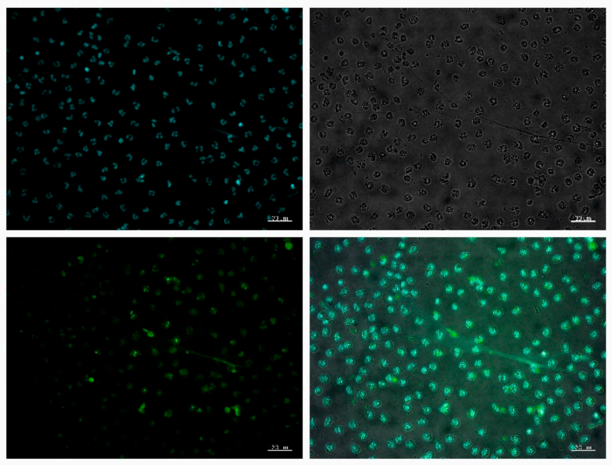

(**b**)

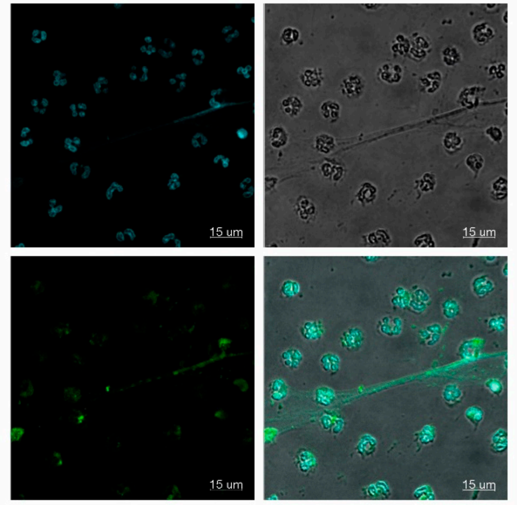


(**c**)

**Figure S2.** Visualization of neutrophil extracellular traps using APF and CB. (**a**)Images obtained by confocal microscopy for preparations of neutrophils incubated with 5 μM APF in the presence of 50 nM PMA; (**b**), (**c**)with 20 μM CB and in the presence of 50 nM PMA. 1 – DAPI (blue), 2 – differential interference contrast, 3 – fluorescence of APF or CB(green) obtained upon excitation withmercury-vapor lamp at 465-505 nm, emission at 515-750 nm (**a**), (**b**) or laser at 488 nm emission at 515-750 nm (**c**), 4 – merge of1,2 and 3.

## References


1. Liu, Z.; Li, G.; Wang, Y.; Li, J.; Mi, Y.; Guo, L.; Xu, W.; Zou, D.; Li, T.; Wu, Y. A Novel Fluorescent Probe for Imaging the Process of HOCl Oxidation and Cys/Hcy Reduction in Living Cells. *RSC Advances*2018, *8*, 9519–9523, doi:10.1039/C7RA13419C.
2. Hu, J.J.; Wong, N.-K.; Lu, M.-Y.; Chen, X.; Ye, S.; Zhao, A.Q.; Gao, P.; Yi-Tsun Kao, R.; Shen, J.; Yang, D. HKOCl-3: A Fluorescent Hypochlorous Acid Probe for Live-Cell and in Vivo Imaging and Quantitative Application in Flow Cytometry and a 96-Well Microplate Assay. *Chemical Science*2016, *7*, 2094–2099, doi:10.1039/C5SC03855C.
3. Jin, L.; Tan, X.; Zhao, C.; Wang, Q. Two Highly Sensitive and Selective Coumarin-Based Fluorometric Probes for the Detection of $ClO^-$ and Cell Imaging. *Analytical Methods*2019, *11*, 1916–1922, doi:10.1039/C9AY00105K.
4. Zhang, R.; Zhao, J.; Han, G.; Liu, Z.; Liu, C.; Zhang, C.; Liu, B.; Jiang, C.; Liu, R.; Zhao, T.; Han, M.-Y.; Zhang Z. Real-Time Discrimination and Versatile Profiling of Spontaneous Reactive Oxygen Species in Living Organisms with a Single Fluorescent Probe. *Journal of the American Chemical Society*2016, *138*, 3769–3778, doi:10.1021/jacs.5b12848.
5. Jiao, C.; Liu, Y.; Pang, J.; Lu, W.; Zhang, P.; Wang, Y. A Simple Lysosome-Targeted Probe for Detection of Hypochlorous Acid in Living Cells. *Journal of Photochemistry and Photobiology A: Chemistry*2020, *392*, 112399–112405, doi:10.1016/j.jphotochem.2020.112399.
6. Choi, M.G.; Lee, Y.J.; Lee, K.M.; Park, K.Y.; Park, T.J.; Chang, S.-K. A Simple Hypochlorous Acid Signaling Probe Based on Resorufin Carbonodithioate and Its Biological Application. *Analyst*2019, *144*, 7263–7269, doi:10.1039/C9AN01884K.
7. Yang, J.; Yao, Y.; Shen, Y.; Xu, Y.; Lv, G.; Li, C. A Novel Phenoxazine-Based Fluorescent Probe for the Detection of HOCl in Living Cells. *Zeitschrift für anorganische und allgemeineChemie*2020, *646*, 431–436, doi:10.1002/zaac.202000127.

8. Bai, X.; Yang, B.; Chen, H.; Shen, J.; Yang, D. HKOCl-4: A Rhodol-Based Yellow Fluorescent Probe for the Detection of Hypochlorous Acid in Living Cells and Tissues. *Organic Chemistry Frontiers* **2020**, *7*, 993–996, doi:10.1039/D0QO00081G.
9. Liu, Y.; Zhao, Z.-M.; Miao, J.-Y.; Zhao, B.-X. A Ratiometric Fluorescent Probe Based on Boron Dipyrromethene and Rhodamine Förster Resonance Energy Transfer Platform for Hypochlorous Acid and Its Application in Living Cells. *Analytica Chimica Acta* **2016**, *921*, 77–83, doi:10.1016/j.aca.2016.03.045.
10. Zheng, W.; Yang, J.; Shen, Y.; Yao, Y.; Lv, G.; Hao, S.; Li, C. The Near-Infrared Fluorescent Probes Based on Phenoxazine for the Rapid Detection of Hypochlorous Acid. *Dyes and Pigments* **2020**, *179*, 108404–108410, doi:10.1016/j.dyepig.2020.108404.
11. Luo, P.; Zhao, X. A Sensitive and Selective Fluorescent Probe for Real-Time Detection and Imaging of Hypochlorous Acid in Living Cells. *ACS Omega* **2021**, *6*, 12287–12292, doi:10.1021/acsomega.1c01102.
12. Das, S.; Aich, K.; Patra, L.; Ghoshal, K.; Gharami, S.; Bhattacharyya, M.; Mondal, T.K. Development of a New Fluorescence Ratiometric Switch for Endogenous Hypochlorite Detection in Monocytes of Diabetic Subjects by Dye Release Method. *Tetrahedron Letters* **2018**, *59*, 1130–1135, doi:10.1016/j.tetlet.2018.02.023.
13. Zheng, D.; Qiu, X.; Chang, L.; Xiaojie Jiao; Song He; Liancheng Zhao; Xianshun Zeng Synthesis and Bioapplication of a Highly Selective and Sensitive Fluorescent Probe for HOCl Based on a Phenothiazine-Dicyanoisophorone Conjugate with Large Stokes Shift. *New Journal of Chemistry* **2018**, *42*, 5135–5141, doi:10.1039/C8NJ00279G.
14. Zhang, Y.; Ma, L.; Tang, C.; Pan, S.; Shi, D.; Wang, S.; Li, M.; Guo, Y. A Highly Sensitive and Rapidly Responding Fluorescent Probe Based on a Rhodol Fluorophore for Imaging Endogenous Hypochlorite in Living Mice. *Journal of Materials Chemistry B* **2018**, *6*, 725–731, doi:10.1039/C7TB02862H.
15. Meng, H.; Huang, X.-Q.; Lin, Y.; Yang, D.-Y.; Lv, Y.-J.; Cao, X.-Q.; Zhang, G.-X.; Dong, J.; Shen, S.-L. A New Ratiometric Fluorescent Probe for Sensing Lysosomal HOCl Based on Fluorescence Resonance Energy Transfer Strategy. *Spectrochimica Acta Part A: Molecular and Biomolecular Spectroscopy* **2019**, *223*, 117355–117360, doi:10.1016/j.saa.2019.117355.
16. Liu, L.; Wei, P.; Yuan, W.; Liu, Z.; Xue, F.; Zhang, X.; Yi, T. Detecting Basal Myeloperoxidase Activity in Living Systems with a Near-Infrared Emissive "Turn-On" Probe. *Anal. Chem.* **2020**, *92*, 10971–10978, doi:10.1021/acs.analchem.9b04601.
17. Lv, J.; Wang, F.; Wei, T.; Chen, X. Highly Sensitive and Selective Fluorescent Probes for the Detection of HOCl/OCl– Based on Fluorescein Derivatives. *Industrial & Engineering Chemistry Research* **2017**, *56*, 3757–3764, doi:10.1021/acs.iecr.7b00381.
