# Supplementary figures and images for: New Application of the Commercially Available Dye Celestine Blue B as a Sensitive and Selective Fluorescent “Turn-On” Probe for Endogenous Detection of HOCl and Reactive Halogenated Species
Source: Antioxidants (Basel). 2022 Aug 30;11(9):1719. doi: 10.3390/antiox11091719 (PMC9495391; doi:10.3390/antiox11091719)

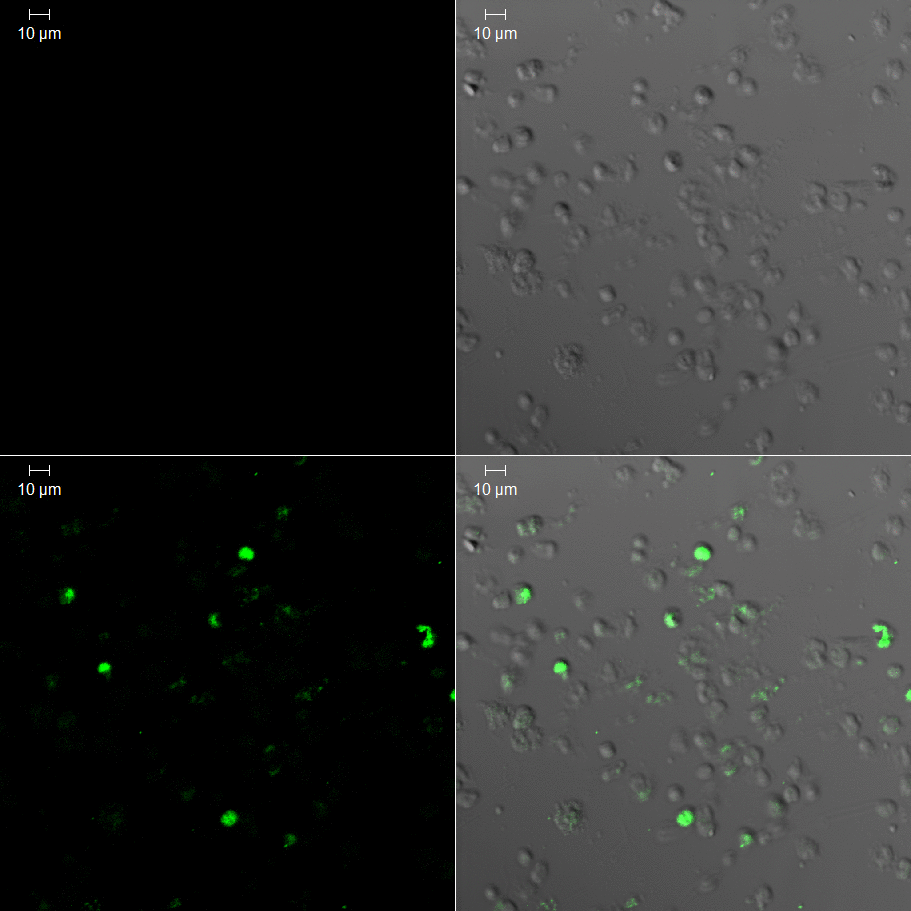

Supplement: Supplementary file 1 [file antioxidants-11-01719-s001.zip › antioxidants-1833452-SM-final/Gif-animations/CB_PMA50.gif]

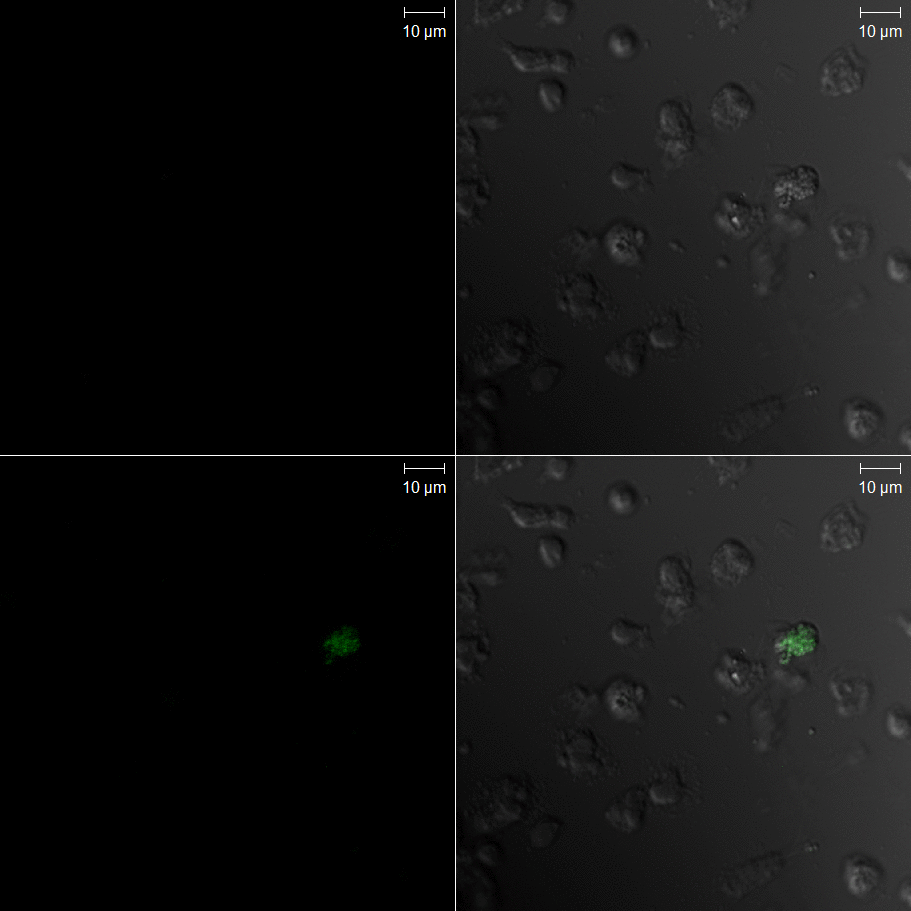

Supplement: Supplementary file 1 [file antioxidants-11-01719-s001.zip › antioxidants-1833452-SM-final/Gif-animations/CB_PMA50_40x.gif]

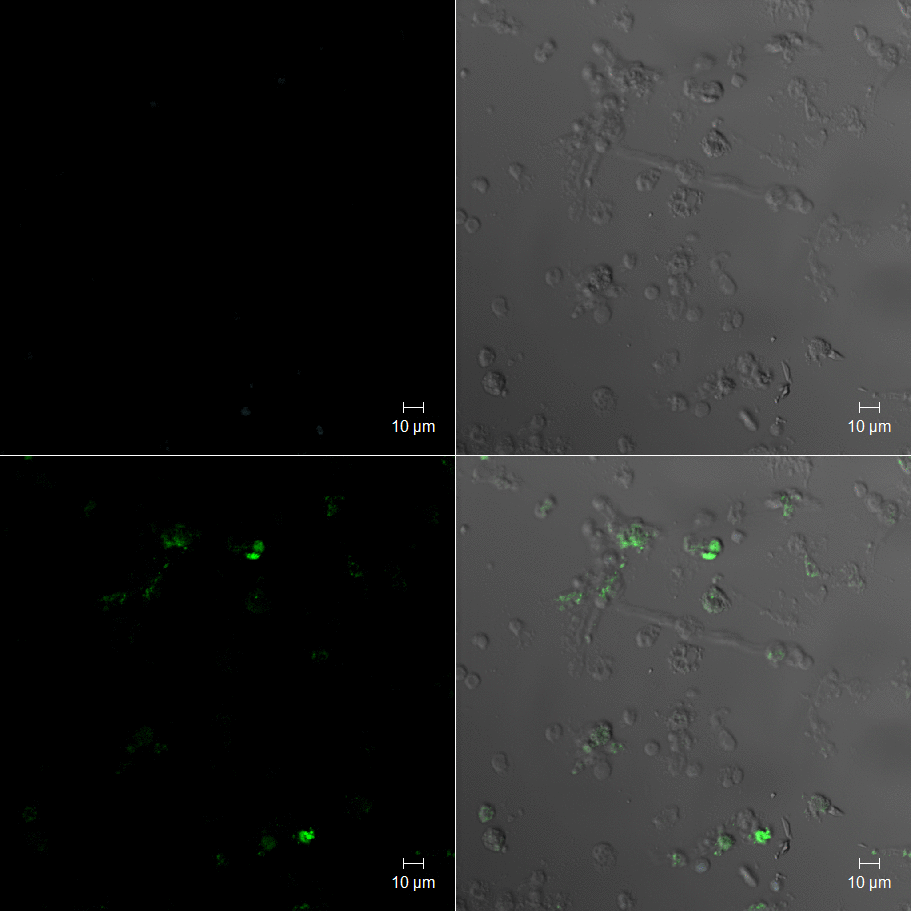

Supplement: Supplementary file 1 [file antioxidants-11-01719-s001.zip › antioxidants-1833452-SM-final/Gif-animations/CB_PMA50_low.gif]

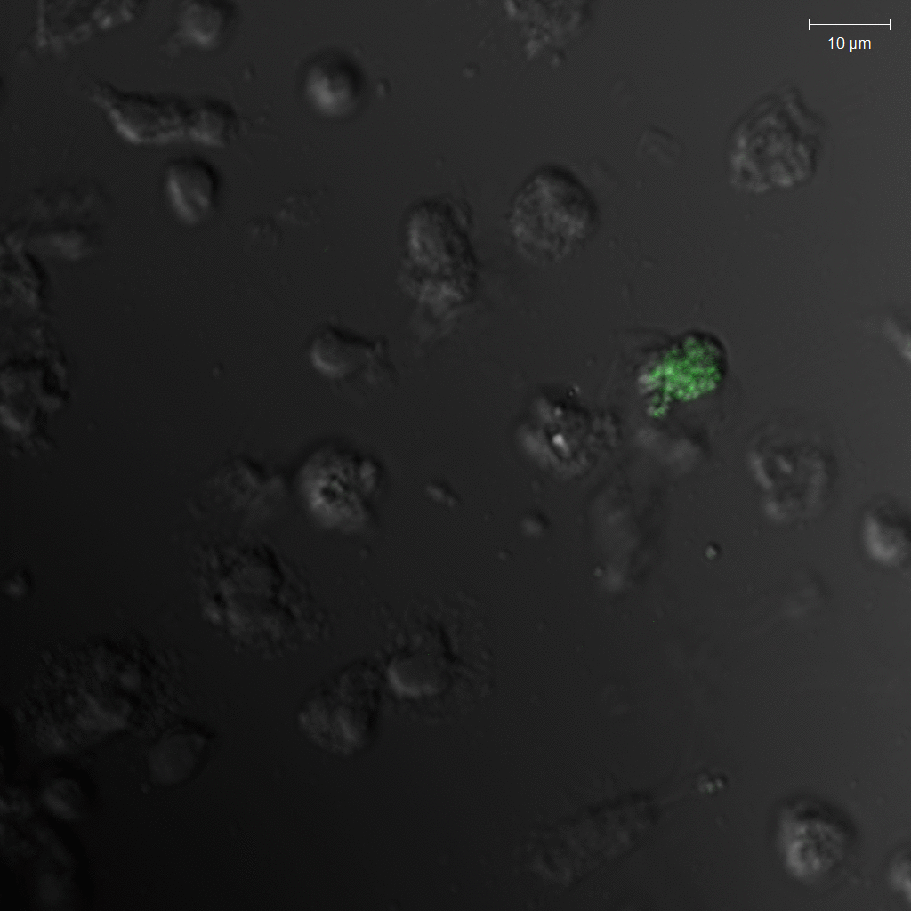

Supplement: Supplementary file 1 [file antioxidants-11-01719-s001.zip › antioxidants-1833452-SM-final/Gif-animations/CB_PMA_40x_comb.gif]

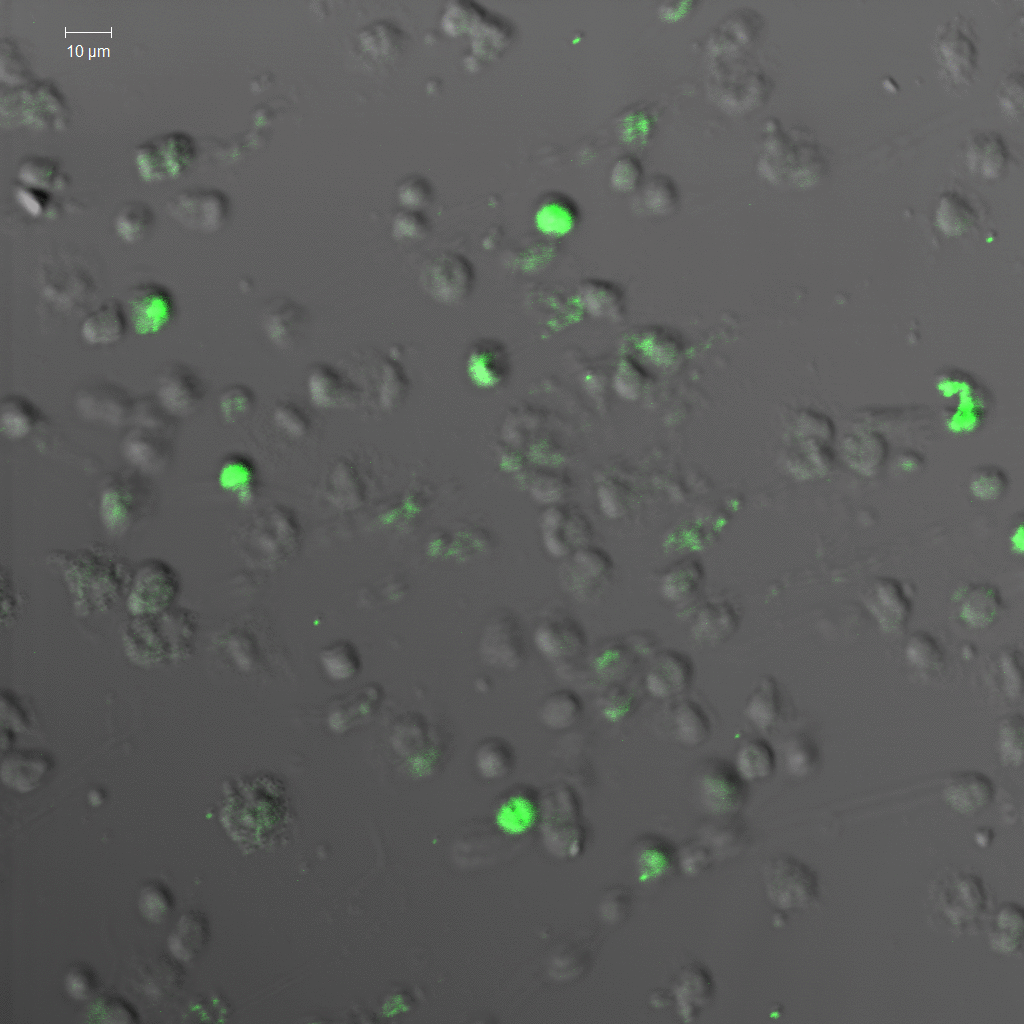

Supplement: Supplementary file 1 [file antioxidants-11-01719-s001.zip › antioxidants-1833452-SM-final/Gif-animations/CB_PMA_combined.gif]

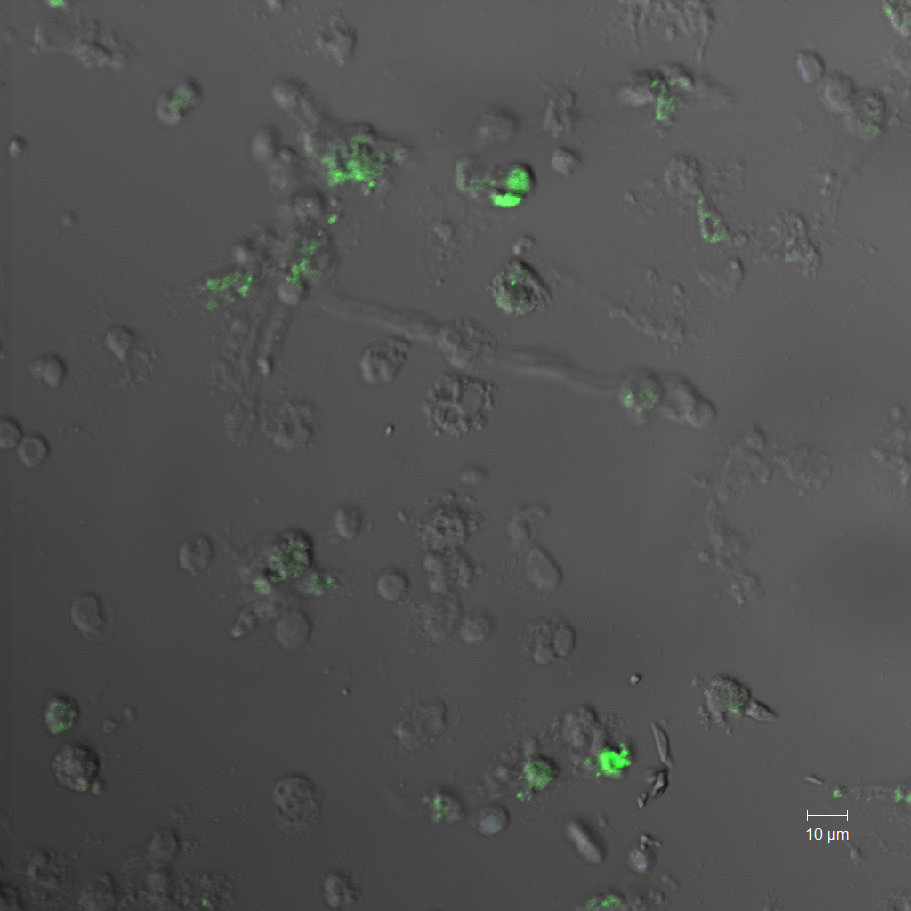

Supplement: Supplementary file 1 [file antioxidants-11-01719-s001.zip › antioxidants-1833452-SM-final/Gif-animations/CB_PMA_low_comb.gif]
